# Prediction of Mental Illness in Heart Disease Patients: Association of Comorbidities, Dietary Supplements, and Antibiotics as Risk Factors

**DOI:** 10.3390/jpm10040214

**Published:** 2020-11-09

**Authors:** Jayanth Sivakumar, Saba Ahmed, Lina Begdache, Swati Jain, Daehan Won

**Affiliations:** 1 Department of Systems Science and Industrial Engineering, The State University of New York at Binghamton, Binghamton, NY 13902, USA; jsivaku1@binghamton.edu; 2 Department of Biological Sciences, The State University of New York at Binghamton, Binghamton, NY 13902, USA; sahmed55@binghamton.edu; 3 Health and Wellness Studies Department, The State University of New York at Binghamton, Binghamton, NY 13902, USA; lbegdac1@binghamton.edu; 4 Department of Computer Science and Engineering, Institute of Technology, Nirma University, Ahmedabad, Gujarat 382470, India; swati.jain@nirmauni.ac.in

**Keywords:** heart disease, risk factors, adjusted odds ratio, mental illness prediction, accuracy, classification, prediction, machine learning, electronic health record

## Abstract

Comorbidities, dietary supplement use, and prescription drug use may negatively (or positively) affect mental health in cardiovascular patients. Although the significance of mental illnesses, such as depression, anxiety, and schizophrenia, on cardiovascular disease is well documented, mental illnesses resulting from heart disease are not well studied. In this paper, we introduce the risk factors of mental illnesses as an exploratory study and develop a prediction framework for mental illness that uses comorbidities, dietary supplements, and drug usage in heart disease patients. Particularly, the data used in this study consist of the records of 68,647 patients with heart disease, including the patient’s mental illness information and the patient’s intake of dietary supplements, antibiotics, and comorbidities. Patients in age groups <61, gender differences, and drug intakes, such as Azithromycin, Clarithromycin, Vitamin B6, and Coenzyme Q10, were associated with mental illness. For predictive modeling, we consider applying various state-of-the-art machine learning techniques with tuned parameters and finally obtain the following: Depression: 78.01% accuracy, 79.13% sensitivity, 72.65% specificity, and 86.26% Area Under the Curve (AUC). Anxiety: 82.93% accuracy, 82.86% sensitivity, 83.35% specificity, and 88.45% AUC. Schizophrenia: 87.59% accuracy, 87.70% sensitivity, 85.14% specificity, and 92.73% AUC. Disease: 86.63% accuracy, 95.50% sensitivity, 77.76% specificity, and 91.59% AUC. From the results, we conclude that using heart disease information, comorbidities, dietary supplement use, and antibiotics enables us to accurately predict the mental health outcome.

## 1. Introduction

Heart Disease is one of the most prevalent conditions in developed countries. It is the leading cause of death in men and women in the United States. Every 37 s, a person dies of Cardiovascular Disease (CVD) [1]. Around 647,000 Americans die of heart disease every year, which is 1 in every 4 deaths. Coronary Artery Disease (CAD) is the most common type of heart disease that killed 365,914 people in 2017. About 18.2 million people in the age group of 20 years and older have CVD [2,3]. Patients with both depression and CVD are three times at risk of mortality than the general population [4,5]. The more severe the depression is, the higher the risk of mortality and further complications from CVD events. The prevalence of depression and anxiety, along with CVD, is bidirectional [6]. This means that the risk of developing CVD in patients with mental illnesses is high, as is the risk of developing depression and anxiety in patients with CVD, which may worsen prognosis [7]. Excess mood disorders and anxiety have been found among people with heart disease irrespective of the countries despite their mental illness prevalence rate [8]. 

Several types of research have been conducted to study the risk of heart disease in patients with mental illnesses. In a study [9] that included 28,734 patients with and without the diagnosed psychotic disorder, it was revealed that those with diagnosed disorders have a significantly higher risk for CVD compared to patients without any diagnosed mental illness. Additionally, in a study aimed to identify CVD risk among veterans, anxiety was associated with CVD mortality in men, and depression was associated with CVD complications among women [10]. Patients with depressive disorders and schizophrenia have an increased risk of developing CHD. Anxiety, along with post-traumatic stress disorder (PTSD), is associated independently with the risk of developing CHD [11,12]. In a case-control study with 3,211,768 patients and 113,383,368 controls, those with schizophrenia had a higher risk for CHD, CVD, and congestive heart failure [13]. 

Among middle-aged women, depression is strongly associated with obesity, lower physical activity, and a high-calorie diet [14]. Obesity increases the risk of developing depression as obese individuals have a higher prevalence of depressive disorders. Additionally, depression was also found to be predictive of obesity [15]. A cross-sectional study on 151,389 patients (age ≥ 18 years) with one or more types of anxiety identified the association of anxiety and hypertension [16]. While in [17], a cross-sectional cohort study on 2028 depressed or anxious patients, the analysis concluded that depression is associated with low systolic blood pressure and less hypertension. However, both hypertension and systolic, as well as diastolic blood pressure, increased with the use of antidepressants. 

Recurrent antibiotic use is associated with a higher risk of depression and anxiety. The study concluded that clarithromycin could induce psychosis manifestations among adult and pediatric patients [18,19]. In addition to that, clarithromycin could induce anxiety, hallucinations, and emotional liability [18,20], while [21] reported dizziness, derealization, and a sense of running very fast with no perception of rotation association with emotional liability, panic-anxiety, and unmotivated crying. In an FDA-sponsored study, clarithromycin showed a 10% increased risk of death from any cause and a 19% increase of risk in developing CVD [22]. Erythromycin associated psychosis was reported in [23]. When a 28-year-old male with schizophrenia conditions was given erythromycin for pityriasis rosea, he suffered from akathisia soon after he took the drug. The literature concluded that akathisia may be induced or precipitated by erythromycin by interfering with other drugs [24]. 

Although several studies have been conducted to identify the risk factors and the bidirectional association of mental illnesses and heart disease, the prediction of mental illness among patients with heart disease in terms of comorbidities, dietary supplement use, and drug usage is sparse. Therefore, this research serves as an exploratory study to identify the risk factors of mental illnesses using comorbidities, dietary supplement use, and antibiotic usage in heart disease patients. In addition to that, the purpose of this research is to accurately predict mental illness (depression, anxiety, and schizophrenia) in heart disease patients and make use of the data-driven model for a real-time prediction system in the health care setting. The datasets in the medical application are not well balanced most of the time, which incurs a highly disproportionate outcome distribution. Our prediction modeling framework deals with the class imbalance problem. Depression, anxiety, and schizophrenia are considered as separate targets, while an aggregate of these three mental illnesses as an outcome for the prediction was also employed in our research.

## 2. Materials and Methods

### 2.1. Variable Selection

Logistic Regression (LR) is a mathematical model that is used to select the statistically significant variables with p-value<0.05. The significant variables are then trained using the methods described below. In addition to that, adjusted $R^{2}$ ($A-R^{2}$) is used for model selection. A good model with useful variables will have a high adjusted $R^{2}$. Adding a useless variable reduces the adjusted $R^{2}$. It is given by $R_{adj}^{2}=1-\left(\frac{\left(1-R^{2}\right)\left(n-1\right)}{n-p-1}\right)$, where n is the number of data samples, p is the number of variables in the model, $R^{2}=1-\frac{D}{D_{0}}$, for a binomial outcome variable. D represents the deviance of the fitted model, $D=-2\log\left(\hat{\beta }\right)$ and $D_{0}$ represents the deviance of the null model, $D_{0}=-2\log\left(\hat{\beta_{0}}\right)$. Bayesian Information Criteria (BIC) select the best subset of variables by penalizing the fitting model based on the number of predictors p. It is the function of the likelihood of the fitted model $BIC=-2\log\left(\hat{\beta }\right)+p\log n$.

Four different types of approaches are implemented: no variable selection and no undersampling; no variable selection but undersampling; variable selection and no undersampling; variable selection and undersampling. For the approaches that involve variable selection, logistic regression that selects the statistically significant variables (p-value<0.05), adjusted $R^{2}$, and BIC are all implemented before the sampling procedure. A similar approach was discussed in [25], where three different approaches were considered in terms of sampling and variable selection. In the literature mentioned, Approach 1 selected the features after sampling, but unsampled data was retained. Approach 2 selected the features after sampling, but the sampled data was retained. Approach 3 sampled after the feature selection. The literature concluded that Approach 1 performed better. In our research, only Approach 3 was assimilated.

### 2.2. Prediction

Machine learning, in its broad-spectrum, learns from the data using any computational algorithm applied to a data sample [26]. The computational model created is used to automatically improve the prediction through pattern recognition or function approximation by using training or historical data. The model created using training data is then tested on the test data set. Some of the model metrics to assess the trained model are explained in detail in the later section. Using these metrics, the best model of interest is chosen for prediction. Machine learning applications are widespread in many kinds of research, which include but are not limited to health care and other clinical studies. 

Some of the machine learning algorithms used in this study for predicting mental illnesses are Random Forest (RF) [27], Decision Tree (DT) [28], Naïve Bayes (NB), Extreme Gradient Boosting (XGBoost) [29], LightGBM (LGBM) [30], and Artificial Neural Networks (ANN) [31]. Random Forest is an ensemble of methods that create a multitude of decision trees at training time for each of the randomly selected bootstrap samples. Using bagging, the Random Forest classifier selects the best split of decision trees among the samples considered during each training step. Prediction on the new data is based on the aggregation of all the decision trees at each step of the training using the majority vote. Decision Tree uses a tree-based classification based on the input features. The non-leaf node represents input features; the leaf node represents target features. Using the information criterion such as Gini Index, Mutual Information, etc., the inputs are split so that they provide the most information, and the target features are classified. The features are recursively split into nodes until the tree reaches a stopping criterion. Naïve Bayes is a probabilistic model that assumes that all the features are independent. The name comes from the naïve assumption that the features are independent. Using conditional probability between the features and the target, the posterior probability is computed based on Bayes’ theorem. Extreme Gradient Boosting is one of the ensemble learning methods that use boosting. This is similar to [32] but is faster and scalable compared to Gradient Boosting Machine (GBM). This algorithm is tree-based learning similar to the Random Forest that uses a distributed gradient boosting technique. Weak learners are added to improve the performance and make it a strong learner. One weak learner might not work well on the data, but the addition of new weak learner will relatively improve the performance. Boosting determines which weak learner should be added next for the given data. This aggregation of weak learners during the training becomes a strong learner and improve model performance. This methodology is implemented in the distributed framework in XGB to make the computation fast and provide accurate predictions. LightGBM is a tree-based learning algorithm similar to XGB. LightGBM has a faster training speed and offers higher accuracy most of the time. This has low memory usage and parallel computing support. This works well on big datasets with larger dimensions and in the cases where the dataset occupies memory. ANN is a neural network model with one or more hidden layers between input and output layers. It approximates classification mathematically for linear and non-linear features. Each layer is fully connected to the previous nodes. Each of the connected nodes is associated with weights. At the training, based on the objective function used, the probability of the weights reaches a certain threshold, and the neurons are fired, which means that the respective fired neurons add more weight in the prediction process.

The dataset is split into a 70% training set and a 30% test set. The dataset is highly imbalanced; therefore, the dataset is undersampled using Synthetic Minority Over-sampling Technique (SMOTE) [33] to match the number of minority classes. With undersampling, the information, as opposed to the original sample size, is still retained. The sampling procedure depends on the approach, which will be covered in the upcoming sections. Classification algorithms are modeled on the training set for four different targets using 5-fold cross-validation. The test set is used for prediction after the model fitting. 

### 2.3. Performance Measure

The aforementioned algorithms are assessed based on accuracy, recall (sensitivity), F1-score, specificity, and Area Under the ROC (Receiver Operating Characteristic) Curve (AUC). True Positive (TP) is the number of correctly classified non-mental illness cases. True Negative (TN) is the number of correctly classified mental illness cases. False Positive (FP) is the number of incorrectly classified non-mental illness cases as mental illness cases. False Negative (FN) is the number of incorrectly classified mental illness cases as non-mental illness cases. Accuracy measures the proportion of correctly classified non-mental and mental illness cases against all the samples. Accuracy is given by $Accuracy=\frac{TP+TN}{TP+FP+FN+TN}$. Recall measures the proportion of actual non-mental illness cases classified correctly against all the non-mental illness case samples. The recall is given by $Recall\left(Sensitivity\right)=\frac{TP}{TP+FN}$. F1-Score measures the average of precision and recall. F1-score is given by $F1\;Score=\left(\frac{2*Recall*Precision}{Recall+Precision}\right)$, where $Precision=\frac{TP}{TP+FP}$, measures the proportion of correctly classified non-mental illness cases against all the classification of non-mental illness cases. If F1-score is high, then both precision and recall is better. Specificity is the measure of correctly classified mental illness cases against all the sample’s mental illness cases. Specificity is given by $Specificity=\frac{TN}{TN+FP}$. ROC measures the True Positive Rate (TPR) against the False Positive Rate (FPR) at different thresholds. The AUC value determines the area under this ROC curve. The higher the value of AUC better the model in terms of differentiating between mental and non-mental illness cases. 

Many researchers inculcated machine learning and artificial neural networks in the health care domain. Machine learning finds its application in a wide range of healthcare-related problem-solving capabilities. Some of the key research has been done in the area of heart disease prediction and mental illness’ association with heart disease. Authors in [34] generated risk prediction models for patients with severe mental illness, such as schizophrenia, along with gender, age, diabetes, Body Mass Index (*BMI*), as well as the use of antidepressants and other antipsychotic drugs. This risk prediction model proved to be better than the Framingham model [35]. For a large population in eastern China with high-risk CVD, a prediction model for a three-year risk assessment was implemented using random forest on 29,930 subjects [36]. The diagnosis of coronary artery disease was conducted using the Classification and Regression Tree (CART) in [37]. In [38], using the Cleveland heart disease dataset from the UCI machine learning repository, coronary artery disease was classified by Naïve Bayes. For the National Health and Nutrition Examination Survey (NHANES) dataset and the Framingham Heart Study CHS dataset, the XGB algorithm was implemented to predict cardiovascular disease [39]. A deep belief network, one of the most common deep learning classifiers, was used to diagnose the Coronary Artery Disease (CAD) using the 24-h ECG signal segments [40].

Python v3.7 and R version 3.6.3 using Jupyter IDE are used for the analysis. Pandas package in Python is used for data wrangling. Preprocessing of data was carried out using *sklearn* package in Python. The adjusted odds ratio is implemented using the package *statsmodels* in Python. The undersampling procedure is implemented using *imblearn* package in Python. Variable selection is implemented using *leaps* package in R programming. The variables selected in R programming are used to select the attributes in the dataframe in Python for creating a prediction model. The prediction models Random Forest, Decision Tree, and Naïve Bayes are created using *sklearn* package in Python. XGBoost is implemented using *xgboost* package in Python. LightGBM is implemented using *lightgbm* package in Python. The artificial neural network structure is created using *keras* package with *TensorFlow* backend in Python. The performance measures are implemented in Python. 

## 3. Results

### 3.1. Dataset

The study protocol was reviewed and approved by the Internal Review Boards of United Health Services (UHS) and Binghamton University. Typically, patients at their first visit undergo a comprehensive medical screening for a history of medical and mental conditions, which is updated at follow-up visits. The dataset was deidentified prior to receipt, including only a few interest variables to the research team. The age-groups were aggregated to minimize the identification of the patients. A de-identified database of 68,647 heart disease patient records was provided by the Cardiology Group at UHS. Upon receipt, the data was stored on the computer of one of the principal investigators with a strong password. The database was only shared with individuals involved directly in the analysis of data. The database included gender, age bracket in 10-year increment, *BMI*, type of heart disease, list of comorbidities and supplement use, laboratory results, antibiotic use, and mental health. Heart diseases include Coronary Heart Disease (CHD), Cardiovascular Disease (CVD), Congenital Disease of Heart (CDH). The data representation for heart diseases is 0 for patients with no heart disease and 1 for patients with that specific heart disease. To better understand the associations, the attributes of CHD, CVD, and CDH are all considered as individual attributes. The categorical attributes are represented by 0 for no and 1 for yes. The mental illness included depression, anxiety, and schizophrenia, which were represented by 0 or 1; in which 1 represents a patient with that specific mental illness. The attributes are encoded and preprocessed using sklearn package in Python 3.7. Table 1 shows the number of observations and percentage distribution of the mental illness and non-mental illness patients segregated by the category of each attribute. A computed column, *Disease,* is added to the dataset as a target. *Disease* is 1 if either *depression* = 1 or *anxiety* = 1 or *schizophrenia* = 1 or if all the three mental illnesses represent 1. It is 0 if all three mental illnesses represent 0. The total number of rows with any one of the mental illnesses (*Disease*) accounts for 32.61%. The non-mental illness patients represent 67.39% of the dataset. The columns *LAB* and *LabValue* contains 97% missing data, while *Gender* had 1 row of unidentified gender listed in the data; therefore, these two columns and one of the unidentified gender rows were removed from the analysis. Of the total 68,646 rows, the number of the missing values in the whole dataset account for 437 rows, of which 434 rows of missing values correspond to the attribute *BMI* and three missing values in the attribute *Gender*. Consequently, a total of 68,209 rows and 29 columns were used for the research. The target columns here are depression, anxiety, schizophrenia, and disease. Table 2 shows the number of observations and the percentage distribution of a combination of mental illnesses. A summary of patients with one or more than one mental illness is listed in the table.

### 3.2. Association of Dietary Supplements, Comorbidities and Drug Usage in Mental Illness Patients

Some of the previous literatures use odds ratio and adjusted odds ratio to identify the risk factors. Psychosis had the largest effect among males and females on CVD among veterans with mental illnesses. The literature used an adjusted odds ratio to determine the effect [10]. Patients with two or more anxiety symptoms had CHD risk and sudden death, which was identified using an adjusted odds ratio [12]. The meta-analysis conducted in [15] showed a reciprocal link between obesity and depression using the odds ratio. A cross-sectional study on 151,389 patients (age≥18 years)  with one or more types of anxiety used final pooled odds ratio and the pooled adjusted hazard ratio to identify the association of hypertension with anxiety [18]. Based on the odds ratio and the associated confidence interval (CI) as well as the *p*-value, suicidal behavior risk factors are identified in [41]. Multivariate logistic regression was used to analyze the association between suicidal behavior and the factors mentioned above. In [18], a case-controlled study was implemented on a medical records database in the UK to study the association of antibiotic exposure on depression, anxiety, and psychosis. Table 3, Table 4, Table 5 and Table 6 shows the odds ratio for depression, anxiety, schizophrenia, and disease. The tables show the number of patients with the mental illness, each attribute’s upper and lower confidence interval under a 95% confidence interval, adjusted odds ratio with its associated p-value. Each of the attributes listed is statistically significant under a 95% confidence interval. The predictors with p-value > 0.05 are excluded from the analysis. The odds ratio listed is an adjusted odds ratio which accounts for confounding variables. The disease represents all three mental illnesses as described in the previous section. Since age is not a continuous attribute, the 10-year increment in the age group is categorized with the age group 0–10 representing a reference level. *BMI* is categorized for the sake of understanding which *BMI* level is a risk factor for mental illnesses. 

### 3.3. Modeling the Mental Illness Prediction Framework

While there was no hyperparameter tuning involved, the model was trained using the default parameter settings for all the algorithms except ANN. For the decision tree, entropy was used as the information criterion. For ANN, the network is designed with 24 neuron-input layers, one hidden layer of 16 neurons, and an output layer. Early stopping criteria were imposed on AUC, which used an Adam optimizer and a Binary Cross Entropy-loss function. A drop-out layer with a probability of 0.5 was used to avoid overfitting. The illnesses are trained as separate targets using random forest, decision tree, Naïve Bayes, XGB, LightGBM, and ANN using 5-fold cross-validation. During variable selection for some of the approaches, the variables are selected using either adjusted $R^{2}$, logistic regression, or BIC before undersampling. Adjusted $R^{2}\;$ and BIC is implemented using R programming, while logistic regression is implemented in python to select the best variable set. After undersampling using SMOTE, the sampled data is split into 5-folds, while in each of the folds, the test set is predicted. At the end of the fold, the reported metrics are the average of the 5-folds. The best performing model is selected based on AUC. The threshold for identifying positive and negative class is 0.5 because the data is balanced using undersampling. The reported values are the average of the 5-folds. The table shows the best performing model using different approaches and the considered algorithms. The comparison of the baseline models and the models trained using the approaches are all reported in the Supplementary material. Table 7 shows a comparison of four approaches for each mental illness. Under the variable selection column in the table, the respective variable selection methodology used is listed, when it is applied. When no such approach is used, it is listed as no. Yes/no undersampling column represent the application of undersampling procedure for the respective model. A model with no variable selection and no undersampling is the baseline model for the targets. The top row in each of the mental illness categories is the best prediction model. 

## 4. Discussion

### 4.1. Risk Factors of Mental Illness

The interpretation of the odds ratio is provided in the supplementary material. In Table 3, age brackets *11*–*20, 21*–*30, 31*–*40, 41*–*50, 51*–*60, 61*–*70,* and >70 are associated with depression. In addition to that, the *female* population has higher odds of depression compared to the *male* population. People who take *Z_pak* are likely to have depression compared to the population that does not take it. With $<18.5\;\mathrm{kg}/m^{2}$ as the reference level for *BMI*, people with *BMI* $\geq 40\;kg/m^{2}$ are likely to be diagnosed with depression. Although other attributes listed like *osteoarthritis*, *coronary artery disease*, *obesity*, and *hypertension* have a lower association with depression, [11,12] mentioned coronary heart disease as one of the risk factors. This partially matched with our analysis. [14,15] concluded that obesity is associated with depression. Although there is no strong association encountered, *obesity* is associated with lower odds of depression. Similarly, *hypertension* has lower odds of depression, as mentioned in [17]. 

In Table 4, the *female* population has higher odds of developing anxiety compared to the *male* population. People who take *clarithromycin, Z_pak,* and *CoQ* have higher odds of developing anxiety. Only the age groups *21–30* and *31–40* are susceptible to developing anxiety. The other predictors listed show lower odds of developing anxiety. *Coronary artery disease* is associated with lower odds of anxiety, which partially match with [11,12]. In addition to that, *hypertension* is associated with lower odds of anxiety, partially matching the conclusions from [16]. *Clarithromycin* is associated with higher odds of anxiety, which agrees with [18,20,21]. 

In Table 5, schizophrenia has a smaller sample size, so the variables selected show association with lower odds of schizophrenia. 

Table 6 represents all three mental illnesses combined. Age groups except for >70 show association of higher odds of developing mental illness. The *Female* population is susceptible to mental illness compared to the *male* population. Drug intake such as *clarithromycin, Z_pak, VitB6,* and *CoQ* is associated with higher odds of developing any of the mental illnesses.

### 4.2. Depression

Compared to the best model (72.65%) the baseline model’s specificity for depression was inaccurate (0.61%). The lower specificity in the baseline model is mainly due to the imbalanced distribution of the classes. The prediction of depression is best with the variable selection that uses adjusted $R^{2}$ and undersampling. LightGBM that uses this approach outperformed on all the metrics compared to the other models. AUC increased to 86.26% in the case of variable selection in comparison to no variable selection, which is 85.18%. The performance of other approaches is provided in the supplementary material. The increase in AUC is obtained after removing *Gender*, *CerebrovascularDisease*, and *elevatedESR*. Having these variables in the model had little to no effect on the prediction. The total number of features in the final model had 21 variables. Since CVD was not one of the predictors of depression, the bidirectional association [6,7] was not concluded in our study. Although CAD is one of the predictors for depression, this inference match with [10].

### 4.3. Anxiety

Specificity for anxiety’s best prediction model using LightGBM (83.35%) is better than the baseline model’s specificity using LightGBM (57.01%). The specificity of anxiety in the baseline model is high in comparison to other mental illnesses because it has a large sample size in the dataset among all the other mental illnesses. There are only two best models that have significantly less difference in performance for the prediction of anxiety. LightGBM and XGB gave AUC of 88.45% and 87.93% respectively. These two were obtained using a variable selection that uses adjusted $R^{2}\;$ and undersampling. The same set of variables emerged similar to the case of depression. LightGBM was selected as the best performing model because it has higher specificity in comparison to XGB. However, the model AUC value was uplifted from 87.75% (no variable selection with undersampling) to 88.45% with the use of the variable selection. Similar to depression, CVD was not selected as a predictor for anxiety from the variable selection. The inference from our research contradicts the bidirectional association in [6,7]. Since CHD and CDH are all selected as a predictor of anxiety, the results match with [11,12].

### 4.4. Schizophrenia

The specificity of the best performing model for schizophrenia is 84.14%, which uses random forest, while the baseline model that uses random forest is 0.74%. Again, it is due to the class imbalance because schizophrenia has a small sample size in the dataset than the other mental illnesses. Schizophrenia’s best prediction model was achieved by selecting the statistically significant variables using logistic regression and undersampling after the selection procedure. A total of seven variables emerged from this method. The selected variables are *Gender, Age, Hypertension, Obesity, CoronaryArteryDisease, BMI*, and *Z_pak*. The variables selected are similar to odd’s ratio interpretation for schizophrenia discussed in Section 4.1. Variables such as gender, age, and *BMI* are used to create the risk prediction model for schizophrenia in [34]. It agrees with the risk prediction model created in our research. Since CAD is selected as one of the predictors of schizophrenia, the results completely agree with [10] and partially match with [13]. The best model’s AUC using random forest is 92.73%, and the case without variable selection is 92.56%.

### 4.5. Disease

For disease, no variable selection with undersampling is the best performing model. Compared to the baseline model’s specificity (56.59%) using lightGBM, the best model’s specificity using LightGBM increased (77.76%). Although there is no significant difference between the AUC values of LightGBM (91.59%) and XGB (91.41%), the specificity for LightGBM (77.76%) is higher than XGB (76.22%). Therefore, LightGBM, with no variable selection and undersampling is selected as the best performing disease prediction model. 

In all cases, the high model performance was achieved through undersampling, because the dataset is highly imbalanced. One of the main advantages of LightGBM is the faster training time on massive datasets. It outperforms other gradient boosting techniques such as XGB as well as the ANN model due to its distributed high-performance gradient boosting technique. This gradient boosting-based method captures all the interactions between the predictors better than linear models. Because the data is well structured, LightGBM performed well for depression, anxiety, and disease. In schizophrenia, since the sample size is smaller, random forest worked better due to the random resampling for training the model. 

The best prediction model for depression is LightGBM, which uses variable selection that uses adjusted $R^{2}$ and undersampling; it gave 78.01% accuracy, 79.13% sensitivity, 72.65% specificity, and 86.26% AUC, respectively. A total of 21 variables emerged for depression from the variable selection. The best model for anxiety is LightGBM, which uses variable selection that uses adjusted $R^{2}$ and undersampling; it gave 82.93% accuracy, 82.86% sensitivity, 83.35% specificity, and 88.45% AUC, respectively. Similar to depression, 21 variables emerged. The best model for schizophrenia is the Random Forest, which uses variable selection that uses logistic regression and undersampling; it gave 87.59% accuracy, 87.70% sensitivity, 85.14% specificity, and 92.73% AUC, respectively. Using logistic regression, schizophrenia has seven variables. The best model for the disease that aggregates all the mental illnesses is LightGBM without using variable selection and uses undersampling; it gave 86.63% accuracy, 95.50% sensitivity, 77.76% specificity, and 91.59% AUC, respectively. Prediction of schizophrenia using our best model was able to achieve high accuracy, followed by a prediction of the disease, anxiety, and depression. The prediction of the disease using our best model accurately captured one of the mental illnesses from their heart disease information, which includes antibiotics and dietary supplement intake.

The healthcare institutions employ an automated prediction system in their diagnostic procedures while using a wide range of data. It is possible due to the increasing innovation in computational efficiency and accurate prediction system. A less time-consuming and accurate prediction of illnesses reduces the cost and time incurred in diagnosing each patient. In this research, a data-driven model to predict mental illness, which uses the information of comorbidities, dietary supplements, and antibiotics, is implemented. This research will find its application mainly to diagnose the illness using comorbidities, dietary supplements, and antibiotics, which is sparse in the medical application to date. The prediction helps identify the likelihood of mental illness based on the intake of drugs, dietary supplements, and antibiotics, along with diagnosed heart disease. This research can be useful when employed in healthcare settings because it helps propose prospective treatment and test procedures that can be time-consuming when done traditionally. 

### 4.6. Strengths and Limitations of the Study

The study has several strengths and limitations. The large number of records studied, and the use of several analytical methods are some of its strengths. One of the limitations of this research is that the patient information comes from only one of the healthcare institutions and does not represent all the patient population. Diagnostic information and patient’s visit to other healthcare institutions outside of this hospital are not known. Also, the patients’ intake of other antibiotics and other dietary supplements for any other purposes other than the aforementioned is not captured in this research. 

## 5. Conclusions 

In summary, patients’ demographics in age groups >10, female population, patients with *BMI* $\geq 40\mathrm{kg}/m^{2}$, and drug intake like Z_pak were associated with depression. The female population, drug intake like clarithromycin, Z_pak, CoQ, and patients’ demographics in age groups 21–30 and 31–40 were associated with anxiety. Z_pak is associated with lower odds of schizophrenia. Patients’ demographics in the age group except >70, who is a female, and drug intakes such as clarithromycin, Z_pak, VitB6, and CoQ were associated with the disease as such, which are a risk factor.

The best prediction model for depression gave 78.01% accuracy, 79.13% sensitivity, 72.65% specificity, and 86.26% AUC, respectively. The best model for anxiety gave 82.93% accuracy, 82.86% sensitivity, 83.35% specificity, and 88.45% AUC, respectively. The best model for schizophrenia gave 87.59% accuracy, 87.70% sensitivity, 85.14% specificity, and 92.73% AUC, respectively. The best model for the disease that aggregates all the mental illnesses gave 86.63% accuracy, 95.50% sensitivity, 77.76% specificity, and 91.59% AUC. We can predict mental illnesses accurately using the dietary supplements, comorbidities, and drug usage data of patients with heart disease.

For future work, the interaction effect of the predictors on depression, anxiety, and schizophrenia will be considered. Applying the odds ratio to these significant interactions and confounders, the risk of each of these interactions, and their effect on mental illness will be interpreted. Since only a few variables are removed instead of selecting a subset of variables for the prediction, traditional subset selection methodologies did not work for the dataset. Therefore, another direction to pursue is to improve the prediction performance by applying Bayesian variable selection approaches. Bayesian net has proved to be useful in many healthcare domains. Applying Bayesian net for mental illness prediction will also be considered as part of the exploration. 

## Figures and Tables

**Table 1:** Summary of drug usage, dietary supplements, and comorbidities for patients with mental illness ^a^.

	Total		Non-Disease		Disease (=Yes)		Depression (=Yes)		Anxiety (=Yes)		Schizophrenia (=Yes)	
Variables	n	%	n	%	n	%	n	%	n	%	n	%
Total	68,209	100.0	45,968	67.39	22,241	32.61	10,080	14.72	15,229	22.24	258	0.38
Gender ^b^												
Male	37,959	44.35	23,552	51.26	6698	30.12	2912	28.89	4445	29.19	150	58.14
Female	30,250	55.65	22,416	48.76	15,543	69.88	7168	71.11	10,784	70.81	108	41.86
Age (Years)												
0–10	487	0.71	237	0.52	250	1.12	25	0.25	229	1.50	0	0.0
11–20	2637	3.87	634	1.38	2003	9.01	858	8.51	1455	9.55	5	1.94
21–30	4641	6.80	1264	2.75	3377	15.18	1312	13.02	2571	16.88	19	7.36
31–40	5647	8.28	2235	4.86	3412	15.34	1402	13.91	2517	16.53	46	17.83
41–50	7727	11.33	4368	9.50	3359	15.10	1521	15.09	2356	15.47	34	13.18
51–60	13,491	19.78	9480	20.62	4011	18.03	1944	19.29	2632	17.28	72	27.91
61–70	14,811	21.71	11,789	25.65	3022	13.59	1556	15.44	1787	11.73	61	23.64
>70	18,768	27.51	15,961	34.72	2807	12.62	1462	14.50	1682	11.04	21	8.14
IDD												
No	67,481	98.93	45,365	98.69	22,115	99.43	10,013	99.34	15,157	99.53	253	98.06
Yes	729	1.07	603	1.31	126	0.57	67	0.66	72	0.47	5	1.94
HT												
No	48,777	28.49	5718	12.44	13,714	61.66	5847	58.01	9739	63.95	152	58.91
Yes	19,433	71.51	40,250	87.56	8527	38.34	4233	41.99	5490	36.05	106	41.09
OA												
No	65,501	96.02	43,933	95.57	21,567	96.97	9714	96.37	14,785	97.08	248	96.12
Yes	2709	3.97	2035	4.43	674	3.03	366	3.63	444	2.92	10	3.88
CM												
No	68,016	99.72	45,808	99.65	22,207	99.85	10,063	99.83	15,209	99.87	258	100.0
Yes	194	0.28	160	0.35	34	0.15	17	0.17	20	0.13	0	0.0
Obesity												
No	59,601	87.38	39,346	85.59	20,255	91.07	9019	89.47	13,974	91.76	226	87.60
Yes	8609	12.62	6622	14.41	1986	8.93	1061	10.53	1255	8.24	32	12.40
CDH												
No	68,153	99.92	45,918	99.89	22,234	99.97	10,076	99.96	15,225	99.97	258	100.0
Yes	57	0.08	50	0.11	7	0.03	4	0.04	4	0.03	0	0.0
HF												
No	66,069	96.86	44,189	96.13	21,879	98.37	9876	97.98	15,005	98.53	256	99.22
Yes	2141	3.14	1779	3.87	362	1.63	204	2.02	224	1.47	2	0.78
CVD												
No	68,115	99.86	45,899	99.85	22,215	99.88	10,063	99.83	15,216	99.91	257	99.61
Yes	95	0.14	69	0.15	26	0.12	17	0.17	13	0.09	1	0.39
AS												
No	68,145	99.90	45,915	99.88	22,229	99.95	10,071	99.91	15,222	99.95	258	100.0
Yes	65	0.10	53	0.12	12	0.05	9	0.09	7	0.05	0	0.0
CAD												
No	59,504	87.24	38,433	83.61	21,070	94.73	9455	93.80	14,523	95.36	245	94.96
Yes	8706	12.76	7535	16.39	1171	5.27	625	6.20	706	4.64	13	5.04
ND												
No	68,121	99.87	45,901	99.85	22,219	99.90	10,075	99.95	15,213	99.989	256	99.22
Yes	89	0.13	67	0.15	22	0.10	5	0.05	16	0.10	2	0.78
E-CRP												
No	67,969	99.65	45,800	99.63	22,168	99.67	10,059	99.79	15,167	99.59	256	99.22
Yes	241	0.35	168	0.37	73	0.33	21	0.21	62	0.41	2	0.78
E-ESR												
No	67,900	99.55	45,757	99.54	22,142	99.55	10,039	99.59	15,154	99.51	258	100.0
Yes	310	0.45	211	0.46	99	0.45	41	0.41	75	0.49	0	0.0
LTUA												
No	67,911	99.56	45,715	99.45	22,195	99.79	10,058	99.78	15,195	99.78	258	100.0
Yes	299	0.44	253	0.55	46	0.21	22	0.22	34	0.22	0	0.0
*BMI*^c^ (mean ± std)	(50.25 ± 1108.60)											
Underweight (<18.5)	1323	1.93	616	1.34	707	3.18	225	2.23	563	3.70	3	1.16
Normal (18.5–24.99)	12,535	18.38	7062	15.36	5473	24.61	2142	21.25	4008	26.32	59	22.87
Overweight (25–29.99)	18,757	27.50	12,939	28.14	5817	26.15	2532	25.12	4052	26.61	84	32.56
Obese (30–39.99)	26,075	38.23	18,720	40.72	7532	33.06	3571	35.43	4803	31.54	88	34.11
Severe Obese (≥40)	9523	13.96	6631	14.43	2892	13.00	1610	15.97	1803	11.84	24	9.30
LAB ^d^												
CRP	595	41.01	382	42.97	213	37.90	87	38.16	170	38.81	0	0.00
ESR	856	58.99	507	57.03	349	62.10	141	61.84	268	61.19	1	100.0
LabValue ^e^ (mean ± std)	(17.79 ± 28.97)											
E_Mycin												
No	68,192	99.97	45,958	99.98	22,233	99.96	10,076	99.96	15,223	99.96	258	100.0
Yes	18	0.03	10	0.02	8	0.04	4	0.04	6	0.04	0	0.00
C_Mycin												
No	67,636	99.16	45,619	99.24	22,016	98.99	9988	99.09	15,066	98.93	257	99.61
Yes	574	0.84	349	0.78	225	1.01	92	0.91	163	1.07	1	0.39
Z_pak												
No	48,024	70.41	33,374	72.60	14,649	65.86	6437	63.86	9969	65.46	201	77.91
Yes	20,186	29.59	12,594	27.40	7592	34.14	3643	36.14	5260	34.54	57	22.09
Folate												
No	68,131	99.88	45,921	99.90	22,209	99.86	10,062	99.82	15,209	99.87	257	99.61
Yes	79	0.12	47	0.10	32	0.14	18	0.18	20	0.13	1	0.39
VitB6												
No	68,050	99.77	45,862	99.77	22,187	99.76	10,054	99.74	15,194	99.77	258	100.0
Yes	160	0.23	106	0.23	54	0.24	26	0.26	35	0.23	0	0.00
CoQ												
No	67,589	99.09	45,490	98.96	22,098	99.36	10,017	99.37	15,137	99.40	258	100.0
Yes	621	0.91	478	1.04	143	0.64	63	0.63	92	0.60	0	0.0
O3FO												
No	68,026	99.73	45,823	99.68	22,202	99.82	10,058	99.78	15,202	99.82	257	99.61
Yes	184	0.27	145	0.32	39	0.18	22	0.22	27	0.18	1	0.39

Abbreviations: AS, Atherosclerosis; *BMI*, Body Mass Index; CAD, Coronary Artery Disease; CDH, CongenitalDiseaseOfHeart; CM, CancerMalignant; CVD, Cardiovascular Disease; C_Mycin, Clarithromycin; CoQ, Coenzyme Q10; E-CRP, Elevated C-reactive Protein; E-ESR, Elevated Erythrocyte Sedimentation Rate; E_Mycin, Erythromycin; HF, HeartFailure; HT, Hypertension; IDD, InsulinDependentDiabetes; LTUA, LongTermUseOfAntibiotics; LV, LabValue; ND, NutritionDeficiency; O3FO, Omega3FishOil; OA, Osteoarthritis; VitB6, Vitamin B-6; Z_pak, Azithromycin; ^a^ Some variable names are abbreviated to display the complete table; ^b^ One unidentified gender and missing data values for three patients (0.006%); ^c^ Body Mass Index (*BMI*) missing data values for 434 patients (0.6%); ^d^ LAB missing data values for 67, 195 patients (97.8%); ^e^ LabValue with miscellaneous values for six patients, <0.3 for 10 patients, <0.1 for three patients, and missing data values for 67, 195 patients (97.8%).

**Table 2:** Summary of non-disease patients and patients with mental illness (*n* = 68,209).

		Disease													
		n						%							
		22,241						32.61							
Non-Disease		Depression		Anxiety		Schizophrenia		(D, A) ^a^		(D, S) ^b^		(A, S) ^c^		(A, D, S) ^d^	
n	%	n	%	n	%	n	%	n	%	n	%	n	%	n	%
45,968	67.39	10,080	14.78	15,229	22.33	258	0.38	3261	32.35	36	0.36	39	0.26	10	0.31

Abbreviations: A, Anxiety; D, Depression; S, Schizophrenia; ^a^ The tuples indicate patients with both depression (D) and anxiety (A). ^b^ The tuples indicate patients with both depression (D) and schizophrenia (S). ^c^ The tuples indicate patients with both anxiety (A) and schizophrenia (S). ^d^ The tuples indicate patients who have anxiety (A), depression (D) as well as schizophrenia (S).

**Table 3:** Association of dietary supplements, antibiotics, and comorbidities for depression.

	N ^a^	LCL ^b^	UCL ^c^	aOR ^d^	*p*-Value
age					
0–10	Ref ^e^				
11–20	858	4.4327	10.2297	6.7335	0.0000
21–30	1312	3.8046	8.7597	5.7730	0.0000
31–40	1402	4.0315	9.2835	6.1178	0.0000
41–50	1521	4.1224	9.4920	6.2551	0.0000
51–60	1944	3.6822	8.4682	5.5840	0.0000
61–70	1556	2.9935	6.8981	4.5440	0.0000
Over–70	1462	2.3545	5.4319	3.5762	0.0000
Gender					
Male	Ref				
Female	7168	1.6113	1.7788	1.6930	0.0000
Z_pak	3643	1.3227	1.4563	1.3879	0.0000
*BMI*					
<18.5	Ref				
>=40	1610	1.0832	1.5228	1.2843	0.0040
Osteoarthritis	366	0.6892	0.8760	0.7770	0.0000
CoronaryArteryDisease	625	0.6284	0.7547	0.6887	0.0000
Obesity	1061	0.4774	0.5561	0.5153	0.0000
Hypertension	4233	0.2373	0.2653	0.2509	0.0000

^a^ Number of patients with depression; ^b^ Lower Confidence Limit; ^c^ Upper Confidence Limit; ^d^ Adjusted Odds Ratio; ^e^ Reference level.

**Table 4:** Association of dietary supplements, antibiotics, and comorbidities for anxiety.

	N ^a^	LCL ^b^	UCL ^c^	aOR ^d^	*p*-Value
Gender					
Male	Ref ^e^				
Female	10,784	1.7604	1.9275	1.8421	0.0000
Clarithromycin	163	1.4329	2.1862	1.7699	0.0000
Intercept		1.4021	2.1573	1.7392	0.0000
Z_pak	5260	1.4481	1.5880	1.5163	0.0000
CoQ	92	1.1736	1.8996	1.4932	0.0011
age					
0–10	Ref				
21–30	2571	1.1618	1.7834	1.4394	0.0009
31–40	2517	1.0576	1.6216	1.3096	0.0134
51–60	2632	0.5436	0.8317	0.6724	0.0003
61–70	1787	0.3454	0.5304	0.4280	0.0000
>70	1682	0.2489	0.3827	0.3086	0.0000
HeartFailure	224	0.6440	0.8746	0.7505	0.0002
*BMI*					
<18.5	Ref				
18.5–24.99	4,008	0.5725	0.7615	0.6603	0.0000
25–29.99	4,052	0.4727	0.6286	0.5451	0.0000
30–39.99	4,803	0.3818	0.5078	0.4403	0.0000
>=40	1,803	0.3542	0.4796	0.4121	0.0000
Osteoarthritis	444	0.5357	0.6772	0.6023	0.0000
CoronaryArteryDisease	706	0.5396	0.6439	0.5894	0.0000
ElevatedCRP	62	0.3983	0.7934	0.5621	0.0011
Obesity	1,255	0.3027	0.3519	0.3264	0.0000
Hypertension	5,490	0.1857	0.2055	0.1954	0.0000

^a^ Number of patients with anxiety; ^b^ Lower Confidence Limit; ^c^ Upper Confidence Limit; ^d^ Adjusted Odds Ratio; ^e^ Reference level.

**Table 5:** Association of dietary supplements, antibiotics, and comorbidities for schizophrenia.

	N ^a^	LCL ^b^	UCL ^c^	aOR ^d^	*p*-Value
Z_pak	57	0.5168	0.9400	0.6970	0.018
Gender					
Male	Ref ^e^				
Female	108	0.3436	0.5766	0.4451	0.0000
Hypertension	106	0.1567	0.2823	0.2103	0.0000

^a^ Number of patients with schizophrenia; ^b^ Lower Confidence Limit; ^c^ Upper Confidence Limit; ^d^ Adjusted Odds Ratio; ^e^ Reference level.

**Table 6:** Association of dietary supplements, antibiotics, and comorbidities for the disease.

	N ^a^	LCL ^b^	UCL ^c^	aOR ^d^	*p*-Value
age					
0–10	Ref ^e^				
11–20	2003	2.0997	3.3290	2.6438	0.0000
21–30	3377	2.4145	3.7744	3.0189	0.0000
31–40	3412	2.1247	3.3040	2.6496	0.0000
41–50	3359	1.5622	2.4222	1.9453	0.0000
51–60	4011	1.0804	1.6695	1.3430	0.0079
>70	2807	0.5097	0.7887	0.6341	0.0000
Intercept		1.8792	2.9404	2.3507	0.0000
Gender					
Male	Ref				
Female	15,543	2.0153	2.1931	2.1024	0.0000
Clarithromycin	225	1.4559	2.1904	1.7857	0.0000
Z_pak	7592	1.5693	1.7137	1.6398	0.0000
VitB6	54	1.0515	2.4383	1.6011	0.0283
CoQ	143	1.2244	1.8736	1.5145	0.0001
*BMI*					
<18.5	Ref				
18.5–24.99	5473	0.5887	0.7924	0.6830	0.0000
25–29.99	5817	0.4823	0.6479	0.5590	0.0000
30–39.99	7352	0.4246	0.5702	0.4921	0.0000
>=40	2892	0.4342	0.5913	0.5067	0.0000
ElevatedESR	99	0.4614	0.8791	0.6369	0.0061
HeartFailure	362	0.5487	0.7188	0.6280	0.0000
CoronaryArteryDisease	1171	0.4861	0.5663	0.5247	0.0000
Osteoarthritis	674	0.4119	0.5139	0.4601	0.0000
ElevatedCRP	73	0.1946	0.3936	0.2768	0.0000
Obesity	1986	0.2164	0.2501	0.2327	0.0000
Hypertension	8527	0.0987	0.1094	0.1039	0.0000
InsulinDependentDiabetes	126	0.0778	0.1216	0.0973	0.0000

^a^ Number of patients with disease; ^b^ Lower Confidence Limit; ^c^ Upper Confidence Limit; ^d^ Adjusted Odds Ratio; ^e^ Reference level.

**Table 7:** Prediction performance of four different approaches for mental illnesses.

Illness	Variable Selection	Under Sampling	Model	Accuracy	F1-Score	Sensitivity	Specificity	AUC
Depression	A-$R^{2}$	Yes	LGBM	0.7801	0.7913	0.8338	0.7265	0.8626
	No	Yes	LGBM	0.7648	0.7745	0.8083	0.7214	0.8518
	No	No	LGBM	0.8530	0.9199	0.9903	0.0615	0.7648
	LR	No	LGBM	0.8524	0.9201	0.9974	0.0160	0.7567
Anxiety	A-$R^{2}$	Yes	LGBM	0.8293	0.8286	0.8251	0.8335	0.8845
	No	Yes	LGBM	0.8242	0.8231	0.8178	0.8306	0.8775
	No	No	LGBM	0.8558	0.9100	0.9380	0.5701	0.8318
	LR	No	LGBM	0.8550	0.9091	0.9331	0.5833	0.8289
Schizophrenia	LR	Yes	RF	0.8759	0.8770	0.8976	0.8514	0.9292
	No	Yes	RF	0.8779	0.8799	0.8983	0.8544	0.9268
	No	No	XGB	0.9962	0.9981	1.0000	0.0000	0.7423
	LR	No	XGB	0.9962	0.9981	1.0000	0.0000	0.7361
Disease	No	Yes	LGBM	0.8663	0.8772	0.9550	0.7776	0.9159
	A-$R^{2}$	Yes	XGB	0.8670	0.8788	0.9646	0.7695	0.9130
	No	No	LGBM	0.8565	0.9035	0.9971	0.5659	0.8522
	LR	No	LGBM	0.8555	0.9028	0.9957	0.5656	0.8515

## Supplementary Materials

The following are available online at https://www.mdpi.com/2075-4426/10/4/214/s1, Table S1: Average Metrics of 5-fold CV for the Mental Illnesses with no Variable Selection and no Under Sampling; Table S2: Average Metrics of 5-fold CV for the Mental Illnesses with no Variable Selection and using Under Sampling; Table S3: Average Metrics of 5-fold CV for the Mental Illnesses with Variable Selection using Adjusted $R^{2}$ and no Under Sampling; Table S4: Variables Selected using Adjusted $R^{2}$ with no Under Sampling; Table S5: Average Metrics of 5-fold CV for the Mental Illnesses with Variable Selection using BIC and no Under Sampling; Table S6: Variables Selected using BIC with no Under Sampling; Table S7: Average Metrics of 5-fold CV for the Mental Illnesses with Variable Selection using Logistic Regression and no Under Sampling; Table S8: Variables Selected using Logistic Regression with no Under Sampling; Table S9: Average Metrics of 5-fold CV for the Mental Illnesses with Variable Selection using Adjusted $R^{2}$ and Under Sampling; Table S10: Variables Selected using Adjusted $R^{2}$ before Under Sampling; Table S11: Average Metrics of 5-fold CV for the Mental Illnesses with Variable Selection using BIC and Under Sampling; Table S12: Variables Selected using BIC before Under Sampling; Table S13: Average Metrics of 5-fold CV for the Mental Illnesses with Variable Selection using Logistic Regression and Under Sampling; Table S14: Variables Selected using Logistic Regression before Under Sampling.
