# Supplementary material for: Prediction of Mental Illness in Heart Disease Patients: Association of Comorbidities, Dietary Supplements, and Antibiotics as Risk Factors
Source: J Pers Med. 2020 Nov 9;10(4):214. doi: 10.3390/jpm10040214 (PMC7712940; doi:10.3390/jpm10040214)
Supplement: Supplementary file 1 [file jpm-10-00214-s001.pdf]

Journal of
Personalized Medicine

Article

Prediction of Mental Illness in Heart Disease Patients: Association of Comorbidities, Dietary Supplements, and Antibiotic as Risk Factors

Jayanth Sivakumar[1], Saba Ahmed[2], Lina Begdache[3], Swati Jain[4], Daehan Won[1,*]

[1] Department of Systems Science and Industrial Engineering, The State University of New York at Binghamton, Binghamton, NY 13902, USA; jsivaku1@binghamton.edu
[2] Department of Biological Sciences, The State University of New York at Binghamton, Binghamton, NY 13902, USA; sahmed55@binghamton.edu
[3] Health and Wellness Studies Department, The State University of New York at Binghamton, Binghamton, NY 13902, USA; lbegdac1@binghamton.edu
[4] Department of Computer Science and Engineering, Institute of Technology, Nirma University, Ahmedabad, Gujarat 382470, India; swati.jain@nirmauni.ac.in
* Correspondence: dhwon@binghamton.edu; Tel.: +1-607-777-5024

Received: 11 September 2020; Accepted: 5 November 2020; Published: 9 November 2020

The source code is available on GitHub.

Odds Ratio Interpretation

The association of the predictors with the mental illnesses are analyzed using the odds ratio (OR). Odds ratio is the measure of how strongly an event is associated with exposure. The ratio is the odds of the event in the exposed group to the odds of the event in the non-exposed group. When

OR=1, exposure does not affect odds of outcome

OR>1, Exposure associated with higher odds of outcome

OR<1, Exposure associated with lower odds of the outcome

This method does not account for controlling confounding variables. The confounding variable's presence affects the results of the variable being studied [1]. The results don't reflect the actual relationship between the exposure and the outcome variable. Logistic regression accounts for multiple confounders. The odds ratio computed from logistic regression's estimates is called the adjusted odds ratio (aOR) because the values are adjusted for the confounders.

Table S1: Average Metrics of 5-fold CV for the Mental Illnesses with no Variable Selection and no Under Sampling.

Illness	Model	Accuracy	F1-Score	Sensitivity	Specificity	AUC
Depression	RF	0.8264	0.8990	0.9066	0.3636	0.7233
	DT	0.8220	0.8963	0.9025	0.3577	0.6413
	NB	0.7154	0.8051	0.7313	0.6236	0.7064
	XGB	0.8523	0.9202	1.0000	0.0007	0.7539
	LightGBM	0.8530	0.9199	0.9903	0.0615	0.7648
	ANN	0.8522	0.9202	1.0000	0.0000	0.7355
Anxiety	RF	0.8143	0.8815	0.8891	0.554	0.7973
	DT	0.8102	0.8791	0.8881	0.5394	0.7256
	NB	0.6812	0.7598	0.654	0.7753	0.7831

	XGB	0.8514	0.9078	0.9416	0.5379	0.8246
	LightGBM	0.8558	0.9100	0.938.0	0.5701	0.8318
	ANN	0.8486	0.9067	0.9469	0.5068	0.8145
	RF	0.9948	0.9974	0.9983	0.0829	0.5756
Schizophrenia	DT	0.9933	0.9966	0.9968	0.0788	0.5411
	NB	0.2136	0.3478	0.2110	0.9098	0.6523
	XGB	0.9962	0.9981	1.0000	0.0000	0.7423
	LightGBM	0.9961	0.9981	0.9999	0.0042	0.7334
	ANN	0.9962	0.9981	1.0000	0.0000	0.6587
	RF	0.8192	0.8676	0.8788	0.6960	0.8395
Disease	DT	0.8187	0.8673	0.8789	0.6943	0.7920
	NB	0.5935	0.6117	0.4776	0.8331	0.7995
	XGB	0.8516	0.8998	0.9891	0.5676	0.8431
	LightGBM	0.8565	0.9035	0.9971	0.5659	0.8522
	ANN	0.8557	0.9033	0.9998	0.5579	0.8394

Table S2: Average Metrics of 5-fold CV for the Mental Illnesses with no Variable Selection and using Under Sampling.

Illness	Model	Accuracy	F1-Score	Sensitivity	Specificity	AUC
	RF	0.7419	0.7504	0.7762	0.7077	0.8098
Depression	DT	0.7424	0.756	0.7984	0.6864	0.7458
	NB	0.6075	0.7155	0.9874	0.2277	0.6116
	XGB	0.7509	0.7578	0.7795	0.7224	0.8359
	LightGBM	0.7648	0.7745	0.8083	0.7214	0.8518
	ANN	0.7443	0.7549	0.7894	0.6993	0.8244
	RF	0.7785	0.7833	0.8006	0.7565	0.8379
Anxiety	DT	0.7722	0.7819	0.8165	0.7280	0.7771
	NB	0.5850	0.7036	0.9852	0.1847	0.5902
	XGB	0.8152	0.8165	0.8222	0.8081	0.8693
	LightGBM	0.8242	0.8231	0.8178	0.8306	0.8775
	ANN	0.8088	0.8121	0.8261	0.7914	0.8589
	RF	0.8779	0.8799	0.8983	0.8544	0.9268
Schizophrenia	DT	0.8683	0.8702	0.8898	0.8423	0.8639
	NB	0.6918	0.7637	1.0000	0.3826	0.6913
	XGB	0.8896	0.8969	0.9636	0.8118	0.9050
	LightGBM	0.8896	0.8950	0.9435	0.8303	0.8977
	ANN	0.7189	0.7523	0.8735	0.5544	0.7443
Disease	RF	0.8366	0.8408	0.8630	0.8102	0.8958
	DT	0.8386	0.8441	0.8740	0.8032	0.8447
	NB	0.8446	0.8632	0.9807	0.7086	0.8507
	XGB	0.8630	0.8756	0.9639	0.7622	0.9141

	LightGBM	0.8663	0.8772	0.9550	0.7776	0.9159
	ANN	0.8551	0.8698	0.9683	0.7418	0.9072

Table S3: Average Metrics of 5-fold CV for the Mental Illnesses with Variable Selection using Adjusted R^2 and no Under Sampling.

Illness	Model	Accuracy	F1-Score	Sensitivity	Specificity	AUC
Depression	RF	0.8271	0.8998	0.9103	0.3479	0.7122
	DT	0.8264	0.8995	0.9114	0.3360	0.6377
	NB	0.7056	0.7939	0.7201	0.6217	0.6963
	XGB	0.8523	0.9202	1.0000	0.0004	0.7408
	LightGBM	0.8527	0.9198	0.9909	0.0565	0.7498
	ANN	0.8519	0.9200	0.9996	0.0001	0.6726
Anxiety	RF	0.8161	0.8829	0.8932	0.5478	0.7887
	DT	0.8128	0.8814	0.8958	0.5239	0.7258
	NB	0.6912	0.7706	0.6746	0.7485	0.7763
	XGB	0.8515	0.9077	0.9404	0.5422	0.8116
	LightGBM	0.8552	0.9094	0.9360	0.5740	0.8189
	ANN	0.8466	0.9056	0.9462	0.5008	0.7998
Schizophrenia	RF	0.9962	0.9981	1.0000	0.0000	0.6917
	DT	0.9962	0.9981	1.0000	0.0000	0.6757
	NB	0.0542	0.0964	0.0507	0.9774	0.5146
	XGB	0.9962	0.9981	1.0000	0.0000	0.7084
	LightGBM	0.9962	0.9981	1.0000	0.0000	0.6676
	ANN	0.9962	0.9981	1.0000	0.0000	0.6721
Disease	RF	0.8190	0.8683	0.8851	0.6825	0.8299
	DT	0.8206	0.8698	0.8894	0.6784	0.7908
	NB	0.5569	0.5550	0.4220	0.8352	0.7903
	XGB	0.8518	0.9000	0.9900	0.5661	0.8292
	LightGBM	0.8556	0.9029	0.9963	0.5649	0.8371
	ANN	0.8548	0.9026	0.9981	0.5586	0.8090

Table S4: Variables Selected using Adjusted R^2 with no Under Sampling.

Depression	Anxiety	Schizophrenia	Disease
age	age	age	age
InsulinDependentDiabetes	InsulinDependentDiabetes	Hypertension	InsulinDependentDiabetes
Hypertension	Hypertension	CancerMalignant	Hypertension
Osteoarthritis	Osteoarthritis	HeartFailure	Osteoarthritis
CancerMalignant	CancerMalignant	CerebrovascularDisease	CancerMalignant
Obesity	Obesity	CoronaryArteryDisease	Obesity
CongenitalDiseaseOfHeart	CongenitalDiseaseOfHeart	NutritionDeficiency	CongenitalDiseaseOfHeart

HeartFailure	HeartFailure	ElevatedCRP	HeartFailure
Atherosclerosis	Atherosclerosis	ElevatedESR	Atherosclerosis
CoronaryArteryDisease	CoronaryArteryDisease	LongTermUseOfAntibiotics	CoronaryArteryDisease
NutritionDeficiency	NutritionDeficiency	Z_Pak	NutritionDeficiency
ElevatedCRP	ElevatedCRP	Folate	ElevatedCRP
LongTermUseOfAntibiotics	LongTermUseOfAntibiotics	CoQ	LongTermUseOfAntibiotics
BMI	BMI		BMI
E_Mycin	E_Mycin		E_Mycin
Clarithromycin	Clarithromycin		Clarithromycin
Z_Pak	Z_Pak		Z_Pak
Folate	Folate		Folate
VitB6	VitB6		VitB6
CoQ	CoQ		CoQ
Omega3FishOil	Omega3FishOil		Omega3FishOil

Table S5: Average Metrics of 5-fold CV for the Mental Illnesses with Variable Selection using BIC and no Under Sampling.

Illness	Model	Accuracy	F1-Score	Sensitivity	Specificity	AUC
	RF	0.8515	0.9196	0.9971	0.0115	0.7423
	DT	0.8518	0.9198	0.9976	0.0109	0.7424
	NB	0.5273	0.6327	0.4846	0.7718	0.7075
	XGB	0.8522	0.9202	1.0000	0.0000	0.7387
Depression	LightGBM	0.8520	0.9200	0.9986	0.0066	0.7401
	ANN	0.8522	0.9202	1.0000	0.0000	0.7263
	RF	0.8548	0.9087	0.9307	0.5907	0.8127
Anxiety	DT	0.8546	0.9086	0.9307	0.5901	0.8122
	NB	0.4211	0.4376	0.2900	0.8773	0.7796
	XGB	0.8515	0.9079	0.9420	0.5366	0.8082
	LightGBM	0.8546	0.9087	0.9310	0.5888	0.8142
	ANN	0.8530	0.9083	0.9371	0.5607	0.8071
	RF	0.9962	0.9981	1.0000	0.0000	0.6515
Schizophrenia	DT	0.9962	0.9981	1.0000	0.0000	0.6515
	NB	0.9962	0.9981	1.0000	0.0000	0.6515
	XGB	0.9962	0.9981	1.0000	0.0000	0.6515
	LightGBM	0.9962	0.9981	1.0000	0.0000	0.6515
	ANN	0.9962	0.9981	1.0000	0.0000	0.6515
	RF	0.8552	0.9025	0.9942	0.5680	0.8352
Disease	DT	0.8555	0.9028	0.9953	0.5668	0.8342
	NB	0.4847	0.4362	0.2958	0.8754	0.7936
	XGB	0.8523	0.9004	0.9909	0.5658	0.8282
	LightGBM	0.8555	0.9028	0.9962	0.5648	0.8366

	ANN	0.8550	0.9026	0.9977	0.5600	0.8282

Table S6: Variables Selected using BIC with no Under Sampling.

Depression	Anxiety	Schizophrenia	Disease
age	Age	Hypertension	age
InsulinDependentDiabetes	InsulinDependentDiabetes		InsulinDependentDiabetes
Hypertension	Hypertension		Hypertension
Osteoarthritis	Osteoarthritis		Osteoarthritis
CancerMalignant	CancerMalignant		CancerMalignant
Obesity	Obesity		Obesity
CongenitalDiseaseOfHeart	CongenitalDiseaseOfHeart		CongenitalDiseaseOfHeart
CoronaryArteryDisease	HeartFailure		HeartFailure
NutritionDeficiency	CoronaryArteryDisease		CoronaryArteryDisease
ElevatedCRP	NutritionDeficiency		NutritionDeficiency
LongTermUseOfAntibiotics	ElevatedCRP		ElevatedCRP
Z_Pak	LongTermUseOfAntibiotics		LongTermUseOfAntibiotics
	Clarithromycin		Clarithromycin
	Z_Pak		Z_Pak
	CoQ		CoQ

Table S7: Average Metrics of 5-fold CV for the Mental Illnesses with Variable Selection using Logistic Regression and no Under Sampling.

Illness	Model	Accuracy	F1-Score	Sensitivity	Specificity	AUC
Depression	RF	0.8518	0.9197	0.9959	0.0205	0.7544
	DT	0.8519	0.9198	0.9963	0.0194	0.7546
	NB	0.5816	0.6903	0.5509	0.7574	0.7201
	XGB	0.8522	0.9202	1.0000	0.0000	0.7524
	LightGBM	0.8524	0.9201	0.9974	0.0160	0.7567
	ANN	0.8522	0.9202	1.0000	0.0000	0.7458
Anxiety	RF	0.8548	0.9087	0.9308	0.5901	0.8260
	DT	0.8547	0.9087	0.9312	0.5884	0.8247
	NB	0.4191	0.4334	0.2860	0.8819	0.7867
	XGB	0.8507	0.9075	0.9426	0.5310	0.8230
	LightGBM	0.8550	0.9091	0.9331	0.5833	0.8289
	ANN	0.8530	0.9084	0.9377	0.5586	0.8190
Schizophrenia	RF	0.9947	0.9973	0.9982	0.0787	0.5665
	DT	0.9934	0.9967	0.9969	0.0788	0.5432
	NB	0.8317	0.9079	0.8330	0.4879	0.6948
	XGB	0.9962	0.9981	1.0000	0.0000	0.7361
	LightGBM	0.9960	0.9980	0.9997	0.0000	0.7340

	ANN	0.9962	0.9981	1.0000	0.0000	0.6681
	RF	0.8548	0.9021	0.9922	0.5710	0.8490
	DT	0.8549	0.9022	0.9930	0.5695	0.8470
Disease	NB	0.4832	0.4309	0.2903	0.8817	0.8008
	XGB	0.8519	0.9001	0.9899	0.5666	0.8433
	LightGBM	0.8555	0.9028	0.9957	0.5656	0.8515
	ANN	0.8551	0.9028	0.9982	0.5594	0.8451

Table S8: Variables Selected using Logistic Regression with no Under Sampling.

Depression	Anxiety	Schizophrenia	Disease
Gender	Gender	Gender	Gender
age	age	age	age
InsulinDependentDiabetes	InsulinDependentDiabetes	Hypertension	InsulinDependentDiabetes
Hypertension	Hypertension	Obesity	Hypertension
Osteoarthritis	Osteoarthritis	CoronaryArteryDisease	Osteoarthritis
CancerMalignant	CancerMalignant	BMI	CancerMalignant
Obesity	Obesity	Z_Pak	Obesity
CongenitalDiseaseOfHeart	CongenitalDiseaseOfHeart		CongenitalDiseaseOfHeart
CerebrovascularDisease	HeartFailure		HeartFailure
CoronaryArteryDisease	Atherosclerosis		Atherosclerosis
NutritionDeficiency	CoronaryArteryDisease		CoronaryArteryDisease
ElevatedCRP	NutritionDeficiency		NutritionDeficiency
LongTermUseOfAntibiotics	ElevatedCRP		ElevatedCRP
Z_Pak	LongTermUseOfAntibiotics		LongTermUseOfAntibiotics
	Clarithromycin		Clarithromycin
	Z_Pak		Z_Pak
	VitB6		Folate
	CoQ		VitB6
			CoQ

Table S9: Average Metrics of 5-fold CV for the Mental Illnesses with Variable Selection using Adjusted R^2 and Under Sampling.

Illness	Model	Accuracy	F1-Score	Sensitivity	Specificity	AUC
Depression	RF	0.7528	0.7633	0.7972	0.7086	0.8189
Depression	DT	0.7539	0.7704	0.8261	0.6817	0.7583
Depression	NB	0.6092	0.7172	0.9913	0.2272	0.6152
Depression	XGB	0.7653	0.7791	0.8278	0.7030	0.8503
Depression	LightGBM	0.7801	0.7913	0.8338	0.7265	0.8626
Depression	ANN	0.7498	0.7622	0.8066	0.6940	0.8289
Anxiety	RF	0.7880	0.7946	0.8200	0.7561	0.8426

Anxiety	DT	0.7815	0.7934	0.8387	0.7244	0.7894
Anxiety	NB	0.5855	0.7039	0.9853	0.1857	0.5949
Anxiety	XGB	0.8224	0.8243	0.8330	0.8118	0.8793
Anxiety	LightGBM	0.8293	0.8286	0.8251	0.8335	0.8845
Anxiety	ANN	0.8098	0.8139	0.8317	0.7878	0.8489
Schizophrenia	RF	0.6027	0.6251	0.6709	0.5326	0.6127
Schizophrenia	DT	0.6047	0.6329	0.6866	0.5206	0.6073
Schizophrenia	NB	0.5115	0.6712	1.0000	0.0235	0.5455
Schizophrenia	XGB	0.6183	0.6267	0.6465	0.5845	0.6307
Schizophrenia	LightGBM	0.6086	0.6493	0.7278	0.4842	0.6278
Schizophrenia	ANN	0.6144	0.6369	0.6901	0.5362	0.6302
Disease	RF	0.8411	0.8467	0.8780	0.8042	0.8912
Disease	DT	0.8446	0.8519	0.8940	0.7953	0.8524
Disease	NB	0.8483	0.8663	0.9830	0.7136	0.8497
Disease	XGB	0.8670	0.8788	0.9646	0.7695	0.9130
Disease	LightGBM	0.8688	0.8795	0.9580	0.7796	0.9128
Disease	ANN	0.8588	0.8716	0.9590	0.7585	0.9013

Table S10: Variables Selected using Adjusted R^2 before Under Sampling.

Depression	Anxiety	Schizophrenia	Disease
age	age	age	age
InsulinDependentDiabetes	InsulinDependentDiabetes	Hypertension	InsulinDependentDiabetes
Hypertension	Hypertension	CancerMalignant	Hypertension
Osteoarthritis	Osteoarthritis	HeartFailure	Osteoarthritis
CancerMalignant	CancerMalignant	CerebrovascularDisease	CancerMalignant
Obesity	Obesity	CoronaryArteryDisease	Obesity
CongenitalDiseaseOfHeart	CongenitalDiseaseOfHeart	NutritionDeficiency	CongenitalDiseaseOfHeart
HeartFailure	HeartFailure	ElevatedCRP	HeartFailure
Atherosclerosis	Atherosclerosis	ElevatedESR	Atherosclerosis
CoronaryArteryDisease	CoronaryArteryDisease	LongTermUseOfAntibiotics	CoronaryArteryDisease
NutritionDeficiency	NutritionDeficiency	Z_Pak	NutritionDeficiency
ElevatedCRP	ElevatedCRP	Folate	ElevatedCRP
LongTermUseOfAntibiotics	LongTermUseOfAntibiotics	CoQ	LongTermUseOfAntibiotics
BMI	BMI		BMI
E_Mycin	E_Mycin		E_Mycin
Clarithromycin	Clarithromycin		Clarithromycin
Z_Pak	Z_Pak		Z_Pak
Folate	Folate		Folate
VitB6	VitB6		VitB6
CoQ	CoQ		CoQ
Omega3FishOil	Omega3FishOil		Omega3FishOil

Table S11: Average Metrics of 5-fold CV for the Mental Illnesses with Variable Selection using BIC and Under Sampling.

Illness	Model	Accuracy	F1-Score	Sensitivity	Specificity	AUC
Depression	RF	0.6979	0.7283	0.8099	0.5862	0.7461
	DT	0.6978	0.7291	0.8137	0.5820	0.7459
	NB	0.5058	0.6689	0.9985	0.0130	0.5061
	XGB	0.6985	0.7270	0.8031	0.5940	0.7414
	LightGBM	0.6966	0.7265	0.8059	0.5876	0.7454
	ANN	0.6959	0.7208	0.7874	0.6045	0.7424
Anxiety	RF	0.7662	0.7934	0.8978	0.6347	0.8167
	DT	0.7655	0.7936	0.9016	0.6295	0.8145
	NB	0.5071	0.6678	0.9907	0.0234	0.5072
	XGB	0.7645	0.7916	0.8948	0.6342	0.8141
	LightGBM	0.7652	0.7930	0.8996	0.6307	0.8172
	ANN	0.7639	0.7950	0.9158	0.6120	0.8089
Schizophrenia	RF	0.6222	0.6339	0.6571	0.5848	0.6210
	DT	0.6222	0.6339	0.6571	0.5848	0.6210
	NB	0.6222	0.6339	0.6571	0.5848	0.6210
	XGB	0.6222	0.6339	0.6571	0.5848	0.6210
	LightGBM	0.6222	0.6339	0.6571	0.5848	0.6210
	ANN	0.6028	0.5143	0.5364	0.6728	0.6235
Disease	RF	0.7852	0.8188	0.9709	0.5995	0.8391
	DT	0.7845	0.8187	0.9733	0.5956	0.8380
	NB	0.7713	0.8075	0.9592	0.5834	0.7951
	XGB	0.7809	0.8139	0.9581	0.6037	0.8351
	LightGBM	0.7853	0.8194	0.9741	0.5965	0.8395
	ANN	0.7821	0.8203	0.9945	0.5698	0.8205

Table S12: Variables Selected using BIC before Under Sampling.

Depression	Anxiety	Schizophrenia	Disease
age	age	Hypertension	age
InsulinDependentDiabetes	InsulinDependentDiabetes		InsulinDependentDiabetes
Hypertension	Hypertension		Hypertension
Osteoarthritis	Osteoarthritis		Osteoarthritis
CancerMalignant	CancerMalignant		CancerMalignant
Obesity	Obesity		Obesity
CongenitalDiseaseOfHeart	CongenitalDiseaseOfHeart		CongenitalDiseaseOfHeart
CoronaryArteryDisease	HeartFailure		HeartFailure
NutritionDeficiency	CoronaryArteryDisease		CoronaryArteryDisease

ElevatedCRP	NutritionDeficiency	NutritionDeficiency
LongTermUseOfAntibiotics	ElevatedCRP	ElevatedCRP
Z_Pak	LongTermUseOfAntibiotics	LongTermUseOfAntibiotics
	Clarithromycin	Clarithromycin
	Z_Pak	Z_Pak
	CoQ	CoQ

Table S13: Average Metrics of 5-fold CV for the Mental Illnesses with Variable Selection using Logistic Regression and Under Sampling.

Illness	Model	Accuracy	F1-Score	Sensitivity	Specificity	AUC
Depression	RF	0.7099	0.7322	0.7935	0.6262	0.7592
	DT	0.7090	0.7325	0.7969	0.6211	0.7546
	NB	0.5072	0.6697	0.9992	0.0152	0.5078
	XGB	0.7076	0.7254	0.7726	0.6429	0.7587
	LightGBM	0.7073	0.7301	0.7917	0.6231	0.7580
	ANN	0.7047	0.7344	0.8168	0.5924	0.7512
Anxiety	RF	0.7719	0.7936	0.8774	0.6665	0.8314
	DT	0.7706	0.7935	0.8819	0.6593	0.8307
	NB	0.5102	0.6697	0.9931	0.0273	0.5105
	XGB	0.7678	0.7905	0.8761	0.6595	0.8289
	LightGBM	0.7697	0.7920	0.8773	0.6622	0.8320
	ANN	0.7647	0.7941	0.9078	0.6215	0.8288
Schizophrenia	RF	0.8759	0.8770	0.8976	0.8514	0.9292
	DT	0.8585	0.8629	0.8976	0.8152	0.8562
	NB	0.6822	0.7537	0.9765	0.3847	0.7429
	XGB	0.8683	0.8758	0.9325	0.7994	0.9234
	LightGBM	0.8838	0.8911	0.9453	0.8179	0.9172
	ANN	0.6374	0.6998	0.8301	0.4531	0.6281
Disease	RF	0.7938	0.8216	0.9493	0.6384	0.8539
	DT	0.7921	0.8206	0.9512	0.6330	0.8527
	NB	0.5095	0.6692	0.9922	0.0268	0.5099
	XGB	0.7835	0.8118	0.9340	0.6329	0.8491
	LightGBM	0.7921	0.8200	0.9475	0.6366	0.8563
	ANN	0.7861	0.8221	0.9886	0.5835	0.8520

Table S14: Variables Selected using Logistic Regression before Under Sampling.

Depression	Anxiety	Schizophrenia	Disease
Gender	Gender	Gender	Gender
age	age	age	age
InsulinDependentDiabetes	InsulinDependentDiabetes	Hypertension	InsulinDependentDiabetes

Hypertension	Hypertension	Obesity	Hypertension
Osteoarthritis	Osteoarthritis	CoronaryArteryDisease	Osteoarthritis
CancerMalignant	CancerMalignant	BMI	CancerMalignant
Obesity	Obesity	Z_Pak	Obesity
CongenitalDiseaseOfHeart	CongenitalDiseaseOfHeart		CongenitalDiseaseOfHeart
CerebrovascularDisease	HeartFailure		HeartFailure
CoronaryArteryDisease	Atherosclerosis		Atherosclerosis
NutritionDeficiency	CoronaryArteryDisease		CoronaryArteryDisease
ElevatedCRP	NutritionDeficiency		NutritionDeficiency
LongTermUseOfAntibiotics	ElevatedCRP		ElevatedCRP
Z_Pak	LongTermUseOfAntibiotics		LongTermUseOfAntibiotics
	Clarithromycin		Clarithromycin
	Z_Pak		Z_Pak
	VitB6		Folate
	CoQ		VitB6
			CoQ

References

1. Pourhoseingholi, M. A.; Baghestani, A. R.; Vahedi, M. How to Control Confounding Effects by Statistical Analysis. *Gastroenterol. Hepatol. from Bed to Bench* **2012**, *5*, 79–83. https://doi.org/10.22037/ghfbb.v5i2.246.

© 2020 by the authors. Submitted for possible open access publication under the terms and conditions of the Creative Commons Attribution (CC BY) license (http://creativecommons.org/licenses/by/4.0/).
